# Supplementary material for: Patterns of Intron Gain and Loss in Fungi
Source: PLoS Biol. 2004 Nov 30;2(12):e422. doi: 10.1371/journal.pbio.0020422 (PMC532390; doi:10.1371/journal.pbio.0020422)
Supplement: Table S1 — Also available at http://genes.mit.edu/NielsenEtAl/. (4.3 MB ZIP). [file pbio.0020422.st001.zip › NielsenEtAl/html/1430.html]

AN1518.1.NCU09804.1.MG06355.1.FG01318.1


```
 CLUSTAL W (1.82) Multiple Sequence Alignments - Introns Inserted


Sequence 1: NCU09804.1	877 aa
Sequence 2: FG01318.1	840 aa
Sequence 3: MG06355.1	880 aa
Sequence 4: AN1518.1	781 aa
Alignment Length: 943 aa
Number Identitical Residues: 213 aa
Alignment Score (without introns) 13447


MG06355.1 	------------~-----------------------------------------MAPTSS
NCU09804.1	MPAPSGQHFQDC2LLSTGDFYVIASSTQPLTYLPLYHDLSAPESFYSSNPARQSMTPTPP
FG01318.1 	------------~-----------------------------------------MTPTPP
AN1518.1  	------------~-----------------------------------------MSPTPP
          	                                                      *:**..

MG06355.1 	STTPSSGGHSPEEQFRVVRKRNRIPLSCTPCRTRK2L2KCDRAHPCGNCVRREGSDTGNC
NCU09804.1	STTSS-GGRSPQEQFRVVRKRNRVPLSCYPCRSRK2L2KCDRNHPCGNCTKRGGTDASSC
FG01318.1 	STNSSQGGRSPEEQFRVVRKRNRVPLSCHPCRTRK2-~KCDRSHPCSNCTKREGMGTSSC
AN1518.1  	STTSSSNGPSPEGQYRIVRKRNRVPLSCGPCRHRK2L~KCNRAHPCENCVKRG--DAHSC
          	**..*..* **: *:*:******:**** *** **   **:* *** **.:*   .: .*

MG06355.1 	SYATPNARSRKNQSQSATTSPDDMQNRIDRLEGLVLALMNGGAADAVASAVATASTAASA
NCU09804.1	FYATPPAR-KKSQCQ-PGASPDDMQNRIDRLEGLVLSLVHGGANIDLPPAATATNAAASA
FG01318.1 	SYATPVSR-KKNQSQ-GDSSPDDMQNRIDRLEGLVLSLMHGGANIDAASAAAAGAAARSA
AN1518.1  	TYAQTNSRKKNSANQAASTSPDDMQNRIDRLEGLVLSLMTNGSQSEGPAAAMAAISGTSS
          	 ** . :*.::.  *:  :*****************:*: .*:    ..*. :  :. *:

MG06355.1 	STASSRAPPSGATPTTSASVTSSGRNDREDYRQSEMMDDDDGDESDIDNGLATSLGVLKV
NCU09804.1	GGHAKGQGASSTTTTSSSSAFLHLDDD------DGPMQDGEG-ESDIDDGLATSLGVLKV
FG01318.1 	-------------TDSGSSAKVEREDE------NAMTYDEEE-NSDEEDGLATSLGFLKV
AN1518.1  	-------------STGSAQHVHDLEIE----------DEGPNQEESETEQVTKSFGIMKM
          	             .  .:.       :           :   .:..  : ::.*:*.:*:

MG06355.1 	DAEKGKAMYIGTESWHTILAG~ISEVKDYFNAHKK~ELESSYEKVLQSKPASAREGPTFL
NCU09804.1	DPDRGKSMYVGQGHWHTVLAD~IAEVKNYFALHKK~DLEKSYEKVMLSKPPTACEGLTFL
FG01318.1 	DTDKGKSLYVGQEHWHTILAD~IS----------E0ELESSYERVKSSKPQAAREGPTLL
AN1518.1  	DNN--KSYYISDAHWASVLSE0ISEVKNYFNTHKK~QYEEQAEKLKATKLPTDVPGSTLL
          	* :  *: *:.   * ::*:  *:. ..     .: : *.. *::  :*  :   * *:*

MG06355.1 	RGAPPAT-EVELRAELPPKSSILTLCGRYFNSMDGAVN~IIHAPTFHAM~LRSHWQDPSK
NCU09804.1	FESTPAA-EIELRAELPSKSTVLTLCGRYFNSMDNAVN~IIHPPTFHQQ~LRNHWLDPSR
FG01318.1 	LGATPAS-EIEIRAELPPKSAVLTLCSRYFNSMDNAVN~IIHGPTFQQQ~LKDHWQDPNK
AN1518.1  	FGAMKATSRAEIMSSFPSKYTTDMLIARYFNCYDPATH1FLHGPTFQAQ0YNKHWEDPSQ
          	  :  *::. *: :.:*.* :   * .****. * *.: ::* ***:    ..** **.:

MG06355.1 	TPIMWIGMLYSILCLAMLSYHKVGDEPTEWKGRSLELASEY1RSRTVQCLVTADYTKPCA
NCU09804.1	TPIMWLGLLYSVMCLAMLSYHKVGDEPPEWKGRTLEKAAEY~RLRTVQCLRVGDYTKPTE
FG01318.1 	TPIMWLGMLYSILCLAMLSYHKVGDEPPEWRGRTLELANEY~RLRTVQCLIKSDYTKPNE
AN1518.1  	TCIVWIGMLFAMMRLAMLSYYREGDEPPEFRGKSLDMAGTF~RNLVAMCLTLADYTKPYP
          	* *:*:*:*:::: ******:: ****.*::*::*: *  : *  .. **  .*****  

MG06355.1 	YTVETMLLYLFGESSSRWDADFGLWLVSSVVTRIAFRMGYHRDGKWFPSITPFHA0EMRR
NCU09804.1	YTVETMILYLFAEHSSRWDADVGLWLIMSQVTRVAFRMGYHRDAKWFPSLTPFQA0EMRR
FG01318.1 	YTVETMILYVFGEYSSRWDADLGLWLIVSLIVRIAFRMGYHRDAKWFPTITPFQA0EMRR
AN1518.1  	YLIECLAFHLHGDFCQTKEADVSVWVLNGVIVRLAMRMGYHRDSKAFPNITPFQG~EMRR
          	* :* : :::..: ..  :**..:*:: . :.*:*:*******.* **.:***:. ****

MG06355.1 	RAWALVKMSDVMFSAQVSLPSMIGEDDFDTELPRNILDDEFGPDIKVLPPSRPRTTPTPI
NCU09804.1	RTWALIRMCDVFFSHQLYLPTMIYEHDCDTDLPHNLADEDFHPDTKVLPPPKPDTEPTNI
FG01318.1 	RTWALVRMSDVIFSHQVSLPSMIYENDCDTQLPNNIFDDEFYPDIKELPPSRPSTVATPI
AN1518.1  	RVWSFVRQADLLFSCQVGLPSMIRMADSDTELPRNLYDDDFDENCKELPPSRPPNEPTPL
          	*.*:::: .*::** *: **:**   * **:**.*: *::*  : * ***.:* . .* :

MG06355.1 	SYMIAKAKLSQEMSNVLKATTLVGRQVSYDDILRLDARLREVVAELPPHLKMQPLDE-SH
NCU09804.1	SYMIAKVKLALELGNILQATGRIRNPVHYDEVLRFDAKLREIKANLPPHLRFQPLSG-CH
FG01318.1 	AYMIAKSRLCNELGNILQATGQVGKHVPYDEIIRFDAKLRQIMQELPPHLKLTTLEG-SH
AN1518.1  	SYLITKSRLANVLGQAIEQTSSLQN-TPYDKVIEMDSELRRARDMIPDHLLVRPFEESSL
          	:*:*:* :*.  :.: :: *  : . . **.::.:*:.**.    :* ** . .:. :. 

MG06355.1 	DPLTLIVARIN~IDVLVQKIFCILHRKYLPRSRENPRYAHSRRTAIEASMDTLRHLETIH
NCU09804.1	DPLTLIIARFN~IDILFQKIICLLHRRYMSKATQNIKYNSSRLRAISSSLESLGHLATLH
FG01318.1 	DPVTLIIARFN~VDILYQKILCLLHRKYLPRARQSPRYAHSRRAAIEASLTALDHLAVLY
AN1518.1  	DPLGLIMSRFS0S--------------------------------------------MLH
          	**: **::*:.                                               ::

MG06355.1 	RESQD-GRLRSLKWFVTSLVTREFLLPAMLIGLDLHYDNQAGSSSQGRHDSHSQHFWTPQ
NCU09804.1	RESQPRGRFRSIKWYVTSIATKDFLLPAMLIVLDLHYDNTLRRT-KTAP---ASPPWTSE
FG01318.1 	RESQSHGRLRSVGWFVKSIATKDFTLPAMLVILDLHFDNIAAQS-AIRQDDEGAVMWDDE
AN1518.1  	VETRPTGRLRSRQNRVTTLSSSDFLQAATIICLDLYHGYNLPSASRASS---DTYAWGRD
          	 *::  **:**    *.:: : :*  .* :: ***:..     ::           *  :

MG06355.1 	QRGQMIRSLEMAQKVWEGLKDTSMEAYKASNAIRIVLEKVKSPHSPSPGSSRENGVSSAS
NCU09804.1	QRAEMIKSLEVTRDIWKGLADESMDALKAFNVLEIMLDKINGTQS-SQHSGSAIEIPTTA
FG01318.1 	QRSKMIGSLETARKIWNTLADTSMEAFKAAKVLDIMLEKIKDPSS-SNADITGVPSPMPG
AN1518.1  	RREEMMAAVQRSKEIWDELRDETMDAWKASGVLGVMLNGLQQRQN----------LEGTP
          	:* :*: ::: ::.:*. * * :*:* **  .: ::*: ::   .             . 

MG06355.1 	VLRGFENNPFGATSSRGETMQDHSAAMTLGMLSGVTPSTTVSPDSVQSPGGTTYRAPPPF
NCU09804.1	TATEF-SDPVQEMRS------EHSAAITLGMLSGSIDASTTSPTHAAQGLDG--PNHPSL
FG01318.1 	LMSGIGADISPPMAP------GFVSPNGLPEFNATANPFSTPNPAAFMGMDFGMPGPEGI
AN1518.1  	VTPAFEPQDE-----------KQSAAMTLGLLSSGMTPIGQSATFNDVSLKPSEPPLPTL
          	    :  :                :.  *  :..   .   .                 :

MG06355.1 	GMDGFGNFG---SGTPAAMGGDMN-MDMFSGF~DMNNN---~TASPFPSFMTNSAQAPGF
NCU09804.1	NMNVS-------ADPATSGTGPLS-MGPTP--~DYLG----~------NTMAGIGDTSPF
FG01318.1 	DFQTDGFAGAGPASPFSSMFGNLG-AGNNAGM~DMAANFDW0NAFENYTQMANWGADQSF
AN1518.1  	GGFGGPETNPGASSIFNNMFGQMPDMQVNLDW0DAWDN---~-----YIQGGTLDTSATW
          	.       ....:.      * : .     .  *   .                     :

MG06355.1 	PANFDW0DAFETYTQTTNFAADSFQ~FLGNNAVEPGTAAAPAEEPDFSFGNQDMSGR
NCU09804.1	ASLFGD~GVGNS---TLDFTTNFDW0FFSGTGPEQSYFSQ-----------------
FG01318.1 	Q-IYGS~GGDQS---SPDQTSSLGG~RSGSTSQGQGIGRRGGGGGGANGTDANMQ--
AN1518.1  	WTPMDI~QQNSG---PLSPSQLPAT~QSNPTERIRTVPRTTNLFPNNGYENDGLT--
          	 :  .     .    . . :        . .              . .  . .
```
